# AI and authorship: Norms and uses to preserve human-led science

**DOI:** 10.1093/pnasnexus/pgag277

**Published:** 2026-09-08

**Authors:** Charles C Branas, Bruce L Levine

**Affiliations:** Department of Epidemiology, Columbia University, New York City, NY 10032, USA; Perelman School of Medicine, University of Pennsylvania, Philadelphia, PA 19104, USA

## A question that is larger than just journal compliance

The use of AI in scientific authorship has moved from a speculative concern to a practical reality. Authors, reviewers, editors, publishers, and readers are now confronting the same question: how can AI be used in ways that improve scientific communication without weakening scientific autonomy, accountability, creativity, or trust?

Current journal policies have begun to converge around several basic norms (1–3). These norms overlap with those adopted by *PNAS Nexus* through Oxford University Press and by other major publishers. Current policies generally agree that AI systems cannot be authors, human authors retain full responsibility, substantive generative use should be disclosed, outputs must be checked, and protected or confidential material should not be entered into insecure systems (4). These are important starting points, but they do not fully address the deeper issue.

More broadly, science is not simply a workflow to optimize. It is an evolutionary system built on independence, serendipity, skepticism, replication, competition, and diverse approaches. Science advances through many independent, often nonlinear and sometimes messy efforts, with selection occurring through evidence, argument, correction, and repeated testing. This distributed structure is what allows science to correct itself and generate novelty. Independence is not incidental to science; it is one of the mechanisms by which reliability and discovery are produced.

For this reason, the use of machine systems to generatively shape scientific output raises a concern beyond whether individual sentences in a manuscript were written by a human or a machine (5). If many investigators increasingly rely on similar systems trained on similar data, scientific outputs will become more correlated, less independent, and more convergent. Simulated diversity within a machine system does not substitute for epistemological diversity across genuinely competing human approaches. Science often thrives on what appears, from a narrow efficiency perspective, to be waste: unexpected findings, failed attempts, divergent paths, and conceptual disagreements. These are not merely costs to be eliminated (6). They are sources of discovery.

## Generative and nongenerative uses of AI are not the same

Blind machine optimization may produce faster iteration and more output but also less exploration of the unexpected. Truly generative advances in science often violate assumptions embedded in prior frameworks and depart from patterns encoded in existing data. These are precisely the advances least likely to emerge from systems trained primarily on the scientific past. Scientific curiosity, question-generation, irony, replication, and critical evaluation must therefore remain human responsibilities, even at the cost of efficiency (7). They are mechanisms through which error is exposed, scientific solutions emerge, and discovery becomes possible.

A useful policy must first distinguish between generative and nongenerative uses of AI. Generative AI creates new content, including text, images, code, predictions, synthetic data, or analytic interpretations, based on patterns learned during training. Generative human intelligence, by contrast, includes the uniquely human work of asking a research question, recognizing an unexplored problem, developing a new method, judging the significance of an unexpected result, and deciding what matters. These forms of scientific intelligence are best left to human activity, human intuition, human accountability, and human creativity.

Nongenerative AI, by contrast, is designed to analyze existing data, recognize existing patterns, classify existing information, or perform constrained operations within predefined rules. In manuscript preparation, nongenerative AI can resemble spellcheck, grammar checking, reference management, statistical software, search engines, or other tools that reduce time spent on rote, nonintellectual tasks. Such tools may change the amount of time spent on mechanical aspects of scientific writing, just as the typewriter, photocopier, personal computer, online search engine, and word processor did before them. Nongenerative AI is like using a jackhammer instead of a pickaxe; generative AI is more like inventing the jackhammer for the first time. They should be treated differently.

## Uses of AI in manuscript authorship

Authors must disclose the application of generative AI, including large language models, chatbots, image generators, code generators, analytic agents, and other generative AI software, when such tools are used during the research or manuscript-preparation process. Disclosure should appear in the Materials and methods section, or in the Acknowledgments if no Materials and methods section is available, and should also be provided during submission when requested. Disclosure should identify the name of the tool, the specific model or version where available, the provider or manufacturer, the date or period of use, and the specific manuscript or research tasks for which the tool was used. AI software may not be listed as an author and cannot assume responsibility for the integrity, originality, accuracy, ethics, or conclusions of the work.

Human authors should be solely accountable for all submitted content, including content generated, revised, analyzed, summarized, translated, coded, visualized, or otherwise assisted by AI. Authors must thoroughly fact-check AI-assisted outputs; verify citations, quotations, data, code, statistical claims, interpretations, and conclusions; ensure that plagiarism, copyright infringement, and misattribution have not occurred; and confirm that all uses comply with applicable ethical, legal, institutional, privacy, and data-security requirements. AI-generated synthetic materials and data must be clearly marked and documented so that they are not mistaken for real-world observations, original human-created images, or unmodified empirical data. Confidential, identifiable, proprietary, embargoed, peer-review, participant, patient, student, institutional, or other protected information must not be entered into public or unsecured AI systems.

Appropriately disclosed use of AI tools will not, by itself, determine editorial assessment or decision-making. However, undisclosed generative AI use, fabricated citations (8), unverified claims, AI-generated imagery presented as empirical observation, breach of confidentiality, or substitution of AI output for human scientific responsibility may constitute grounds for editorial concern, correction, rejection, retraction, or broader forensic scrutiny.

Allowable uses of AI include tasks in which the machine assists, organizes, accelerates, checks, translates, formats, or improves work while leaving scientific judgment, accountability, and creativity with human authors. These may include hypothesis generation; literature search, review, synthesis, and gap determination; analysis of market trends or patent environments; research design support; development of experimental or research protocols; selection of research methods; code generation, optimization, and debugging; process automation; creation of algorithms for data analysis; secondary data collection; data validation, cleaning, curation and organization; data analysis and visualization; reproducibility testing; proofreading and editing; summarizing text; language translation; reformatting; and preparation of press releases or outreach summaries (7, 9, 10). These allowable uses should be disclosed when they involve generative AI or creatively affect the submitted research or manuscript. For example, AI and machine learning may be used retrospectively to analyze patient, manufacturing, and response data to predict early biomarkers of response to CAR T cell therapy (11). AI models may also be used prospectively, for instance in analyzing publicly available datasets to nominate CAR T cell targets for tumors subsequently validated by human scientists in experimental models. The latter is also a worthwhile example because disclosure of AI use is clearly indicated in the title of the manuscript itself (12).

Nonallowable uses of AI are those in which a machine replaces human scientific responsibility for the core intellectual, creative, ethical, interpretive, or accountability-bearing work of research. These tasks are to be performed by human authors and not delegated to a machine or generative AI system. They include initial conceptualization and idea generation; defining the original research objective; seeding research questions; feasibility assessment and risk evaluation; evaluating the human utility of the research; primary person-to-person data collection; formulating final conclusions; bias analysis and assessment of potential discrimination; ethical risk analysis and monitoring compliance with ethical standards; data-confidentiality monitoring; quality assessment of data or writing; identification of the full universe of limitations; and recommendations for final policies or actions based on results (7, 10). Our proposed list is consistent with prior AI use safeguards that many journals have adopted but goes beyond them in some respects, notably that human authors should critically construct and own their research questions. The scientific publishing community should seek to ensure that the originating research question and final evidentiary judgment in any published manuscript remain human-led. This should be a human innovation, if for no other reason, than allowing machines to create and seed initial research questions could put us in a downward spiral of over-derivative redundancy given that machines fundamentally recycle the information and knowledge upon which they have been trained.

The distinction between allowable and nonallowable uses of AI is not a distinction between minor and major tasks, or between easy and difficult tasks (13). It is a distinction between assistance and authorship, between tool use and creative delegation, between machine support and human responsibility. AI may help authors work more carefully, more clearly, more efficiently, and more reproducibly. It may not replace the human autonomy, accountability, judgment, and creativity that make science trustworthy.

## A norm of augmentation without abdication

Our aim should not be to conduct science without AI. AI can help researchers navigate an exponentially growing scientific literature, work across languages, detect errors, improve reproducibility, support increasingly complex analyses, and communicate findings more effectively. Refusing these benefits is neither realistic nor desirable. The more durable principle is one of augmentation without abdication: using AI to extend human capacity without surrendering scientific autonomy, independence, or responsibility. Human control must mean more than providing final approval to a process largely directed by machines. It must include meaningful human authorship of the questions asked, choices made, interpretations offered, and conclusions drawn.

A scientific manuscript is more than a refined record of results. It documents an inevitably imperfect chain of human choices about what mattered, what was observed, what was tested, what was believed, and what remained uncertain. AI can support nearly every link in that chain, but it should not render the chain authorless. The current moment therefore requires a reaffirmation of what manuscript authorship means: a scientific paper is not only a report of findings, but a record of human judgment applied to evidence. AI can help prepare that record, but it cannot be responsible for it. The future of scientific publishing should not be organized around maximizing machine-assisted output alone. It should preserve the independence, redundancy, inefficiency, criticism, imagination, ethical reflection, and human curiosity through which science discovers what we do not already know.
